# Quality-of-Life and Weight Loss Outcomes Following an Individualized Nutrition and Physical Activity Counseling Program in Breast Cancer Survivors: A Prospective Single-Arm Pilot Study

**DOI:** 10.3390/cancers18193198

**Published:** 2026-10-03

**Authors:** Shashi Kant, Ahmad Alhalabi, Nadeem Bilani, Iktej Singh Jabbal, María Herrán, Theresa Abdo, Saad Sabbagh, Kaylee Sarna, Amanda Stevenson, Diana Saravia, Candice Schwartz, Thomas Samuel, Zeina Nahleh, Elizabeth Stone

**Affiliations:** 1 Department of Hematology-Oncology, Maroone Cancer Center, Cleveland Clinic Florida, Weston, FL 33331, USAstevena7@ccf.org (A.S.);; 2 Department of Clinical Research, Cleveland Clinic Florida, Weston, FL 33331, USA

**Keywords:** breast cancer survivors, weight loss, quality of life, nutritional program

## Abstract

Purpose: Assess the feasibility of a 6-month individualized nutrition counseling and exercise program targeting a 10% body weight loss in breast cancer (BC) survivors, with secondary endpoints assessing metabolic parameters, cardiovascular function, and quality of life. Methods: This single-arm pilot study enrolled BC survivors with body mass index (BMI) ≥ 25 kg/m^2^ in a counseling program with a registered dietitian, consisting of in-person and telephone visits over six months. Outcomes measured included weight, body composition, blood markers, cardiovascular function, and quality of life at 3 and 6 months. Results: A total of 78 breast cancer survivors were enrolled; 59 remained in the study at Month 3, and 54 completed the 6-month follow-up. The median age of participants was 58.5 years, and the cohort was predominantly White (72.4%). Among the 54 participants who completed 6-month follow-up, 11.1% achieved >10% weight loss and 29.6% achieved >5% weight loss. At 3 months, significant reductions were observed in BMI, body fat composition, and hemoglobin A1c (HbA1c), along with improvements in quality of life, depression, and anxiety. At 6 months, significant reductions were observed in weight, BMI, and body fat composition, with improvements in quality of life and depressive symptoms. Conclusions: This hybrid nutrition and exercise program was associated with observed improvements in weight loss and improved quality of life among BC survivors. Implications for Cancer Survivors: A low-burden, hybrid nutrition and exercise program integrated into routine oncology follow-up may help breast cancer survivors achieve weight loss while improving their quality of life.

## Simple Summary

Breast cancer survivors frequently experience weight gain, unfavorable body composition changes, and reduced quality of life following treatment. We evaluated a six-month hybrid nutrition and physical activity counseling program delivered by a registered dietitian through a combination of in-person visits and telephone follow-up. Participants demonstrated improvements in weight-related outcomes, quality of life, and depressive symptoms. Importantly, the intervention required relatively few patient contacts compared with more intensive lifestyle intervention programs, potentially facilitating integration into routine survivorship care. Responses varied among participants, highlighting the need for more personalized approaches to weight management after cancer treatment. These findings support the feasibility of low-burden lifestyle interventions and provide preliminary evidence that such programs may contribute to improved health and well-being among breast cancer survivors.

## 1. Introduction

Breast cancer (BC) remains the most diagnosed cancer and the second leading cause of cancer-related mortality among women in the United States (U.S.) [1]. Due to advancements in treatment and earlier diagnosis, the number of BC survivors has significantly increased, with nearly 4.0 million BC survivors in the U.S. [2]. While increased survivorship is encouraging, the long-term effects of cancer and its treatment present significant health challenges and represent a growing public health concern.

Obesity is more common among BC survivors than in the general population. Between 50% and 96% of survivors gain weight during treatment [3,4,5,6], and a substantial proportion of survivors experience progressive, sustained weight gain that persists into the months and years following primary treatment, rather than resolving once treatment ends [3,4,5,6]. This weight gain is strongly associated with chemotherapy, which varies according to the chemotherapeutic regimen [7], duration [8], and treatment-related factors (e.g., fatigue and taste changes) [9]. Additionally, changes in patient behavior, including reduced physical activity and dietary modifications, further exacerbate weight gain. Moreover, high body mass index (BMI) has been associated with an increased risk of BC recurrence [10], specific mortality [11], and all-cause mortality [12].

In addition to weight gain affecting prognosis, health-related quality of life in BC survivors is negatively impacted by being overweight or obese. As a result, patients report poorer quality of life after BC treatment compared to assessments made at the time of diagnosis [13]. Beyond the impaired body composition and inflammatory markers, BC survivors also experienced worsening of pain [14], anxiety [15], depressive symptoms [15], and body-image perception [16] following treatment.

Prior controlled trials, including LEAN and LISA, have established that structured dietitian-led counseling can produce clinically meaningful weight loss in breast cancer survivors, but these trials were generally delivered through dedicated, well-resourced study infrastructure with intensive contact schedules. Considerably less evidence exists on whether a lower-intensity, structured model delivered within routine oncology follow-up, more representative of what most survivorship clinics could realistically implement, may achieve similar engagement and outcomes. This gap in real-world, resource-proportionate evidence, rather than efficacy under ideal trial conditions, is the specific rationale for the present study.

Although the American Cancer Society endorses weight gain prevention and recommends nutrition and physical activity programs for patients with BC during and after therapy [17], less than one-third participate in the recommended levels of physical exercise [18,19]. In-person counseling is the standard approach for weight loss interventions, but it may incur travel costs, limiting participation, adherence, and long-term sustainability. Therefore, we aimed to evaluate the feasibility of a 6-month individualized nutrition counseling and exercise program designed to achieve >10% weight loss and assess its association with changes in inflammatory markers and quality of life in BC survivors.

## 2. Methods

### 2.1. Study Design

**Figure 1:**
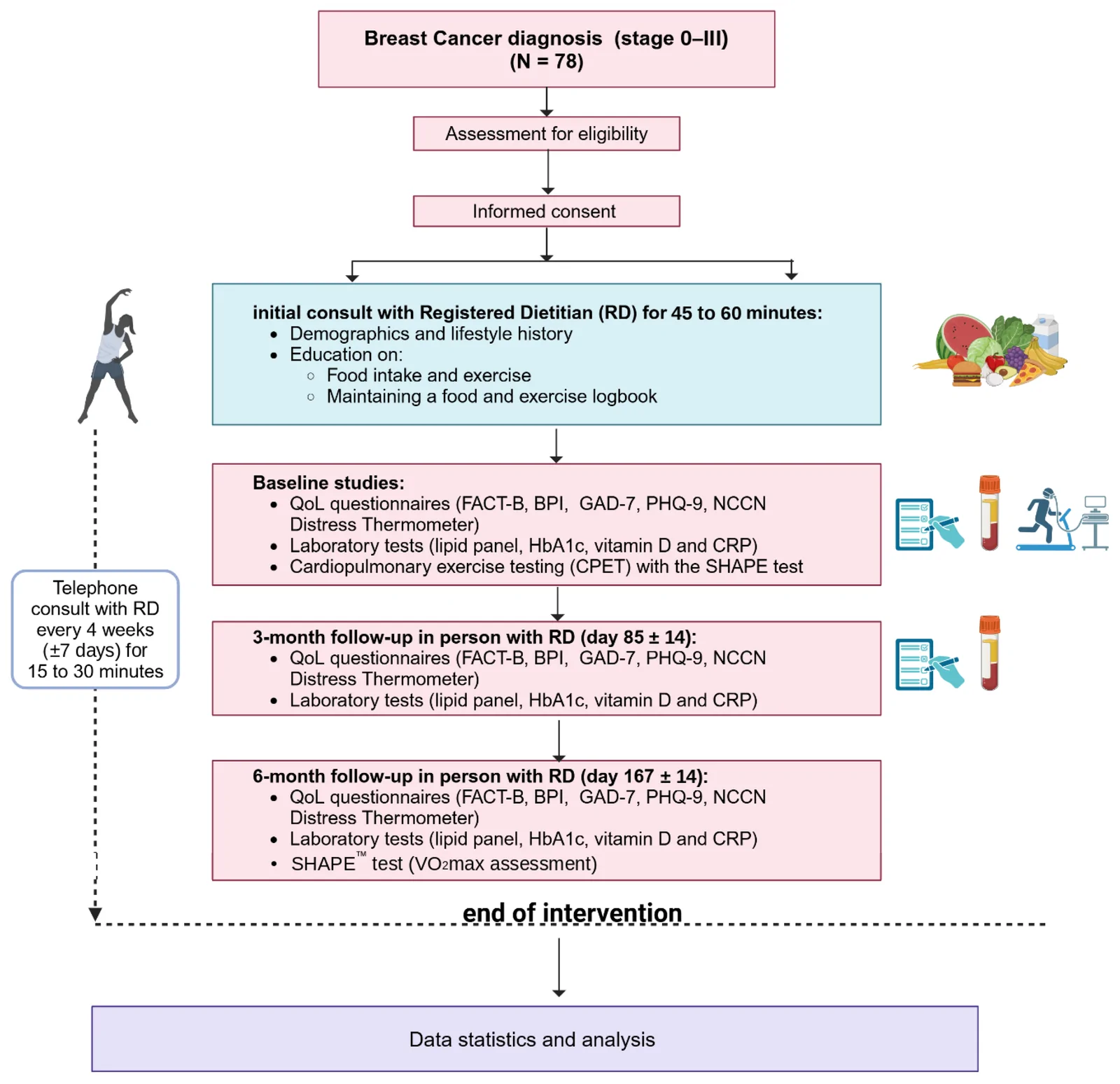
Flow chart of the pilot study design based on the standard protocol. Solid arrows indicate the sequence of study procedures; the vertical dashed arrow indicates the period of telephone consultations with the registered dietitian (RD), and the horizontal dashed line marks the end of the intervention.

This prospective, single-arm pilot study was conducted at Cleveland Clinic Florida from October 2021 to November 2023. The study design is illustrated in Figure 1. Participants attended in-person visits with a registered dietitian at baseline, 3 months, and 6 months, with telephone nutrition consultations every 4 weeks to assess compliance and provide counseling. Quality-of-life questionnaires and laboratory tests were completed at baseline, 3 months, and 6 months (Table 1). The study was registered on ClinicalTrials.gov (NCT04365569; first posted on 28 April 2020).

The study protocol was approved by Cleveland Clinic Florida Institutional Review Board (CASE 11119), and written informed consent was obtained from all participants before enrollment.

### 2.2. Patient Characteristics

Eligible participants were: (1) women ≥ 18 years; (2) Stage 0–III BC; (3) BMI ≥ 25.0 kg/m^2^; and (4) undergoing follow-up by the oncology team. Exclusion criteria included active treatment with chemotherapy, immunotherapy, or radiation therapy. Participants could take selective estrogen receptor modulators (SERMs), or aromatase inhibitors (AIs) during the study.

### 2.3. Suspension Criteria

Patients were discontinued from the study if they met any of the following conditions: (1) progression to stage IV; (2) comorbidities preventing exercise or dietary participation; (3) safety concerns, as determined by the investigator; (4) adverse events; (5) pregnancy; or (6) mortality.

### 2.4. Intervention

The intervention included an initial in-person consultation with a registered dietitian (RD), lasting 45–60 min, focused on two main components: (1) nutritional and (2) physical activity counseling. Additionally, in-person follow-ups were scheduled at 3 and 6 months, with monthly 15 to 30-min phone consultations, except during months with in-person visits.

Counseling was individualized based on each participant’s baseline anthropometrics, dietary habits, lifestyle factors, and clinical characteristics. During follow-up interactions, the RD reviewed adherence, addressed barriers to implementation, and adjusted nutritional and physical activity recommendations accordingly.

#### 2.4.1. Nutritional Counseling

A single RD provided one-on-one dietary counseling to all participants, following the American Cancer Society Nutrition and Physical Activity guidelines [17]. The focus was on three main components: (1) limiting processed and red meat intake to <6 ounces per week; (2) consuming at least 2.5 cups of vegetables and fruits each day; (3) replacing refined grains with whole grains, with full compliance defined as >75% consumption. Diet quality was assessed using a predefined diet quality scoring system based on American Cancer Society dietary recommendations. Each of the three dietary components was scored from 0 to 3 according to predefined protocol criteria, resulting in a total score range from 0 to 9. Participants received educational materials and a Daily Food and Exercise Log. Counseling sessions were tailored to participant-specific needs and reinforced during follow-up visits to optimize adherence.

Dietary intake was assessed through the RD-led clinical counseling process rather than a separate validated dietary-intake instrument: no 24-h dietary recall, food-frequency questionnaire, or structured multi-day food diary was administered for research purposes. Caloric requirements were estimated by the RD as part of routine counseling to individualize recommendations, and eating-out frequency was tracked as one of the counseling variables; neither constitutes a quantitative baseline dietary intake measurement, which was not separately collected in this study.

#### 2.4.2. Physical Activity Counseling Program

Participants were educated on physical activity guidelines based on recommendations from the U.S. Department of Health and Human Services and the American Cancer Society [20]. The program recommended either 150 min of moderate- or 75 min of vigorous-intensity physical activity (or a combination of both) per week over six months. Activity was self-reported during monthly phone calls or in-person visits at months 3 and 6. The RD also recorded weekly exercise duration (in minutes) and metabolic equivalent (MET) scores for each activity using the Ainsworth guidelines [21]. Physical activity recommendations were tailored to individual tolerance, baseline activity levels, and patient-reported limitations.

### 2.5. Measure of the Intervention

#### 2.5.1. Weight Loss and Body Fat Percentage

The RD measured weight and body fat percentage at baseline, 3 months, and 6 months using bioelectrical impedance analysis, a non-invasive method in which a current passes between two surface electrodes placed on the right hand and right foot [22].

#### 2.5.2. Quality of Life Assessment

Participants completed validated surveys at baseline, 3 months, and 6 months:

##### FACT-B

The Functional Assessment of Cancer Therapy for Breast Cancer (FACT-B) is a validated patient-reported outcome questionnaire assessing quality of life in patients with BC [23]. It includes 36 items across five domains: physical well-being, social/family well-being, emotional well-being, functional well-being, and a BC-specific subscale. Higher scores indicate better quality of life (0–4 scale score).

##### Pain

The Brief Pain Inventory Short Form (BPI-SF) is a validated, self-reported questionnaire assessing pain severity and interference [24]. The severity subscale evaluates pain in four states (worst, least pain, average, and current pain over the past 24 h). Each is rated on a 0–10 scale, with higher scores indicating greater pain; the total score is the average of the four items.

The pain interference subscale evaluates the impact of pain on seven daily activities (general activity, walking, work, mood, enjoyment of life, relations with others, and sleep), also rated on a 0–10 scale. The interference score is the mean of these items.

##### Anxiety

The Generalized Anxiety Disorder 7-item (GAD-7) scale is a validated questionnaire to assess anxiety symptoms [25], with a total score ranging from 0 to 21.

##### Depressive Symptoms

Depressive symptoms were evaluated using the Patient Health Questionnaire-9 (PHQ-9) [26]. Participants rated the severity of symptoms experienced over the past two weeks on a scale ranging from 0 to 27.

##### Distress

The National Comprehensive Cancer Network (NCCN) Distress Thermometer is a single-item screening tool to identify patients experiencing distress [27]. A score of 5 or higher indicates moderate to severe psychological distress.

#### 2.5.3. Correlative Markers

Each participant was prescribed a panel of blood tests, including triglycerides, low-density lipoprotein (LDL), high-density lipoprotein (HDL), total cholesterol, hemoglobin A1c (HbA1c), 25-hydroxyvitamin D, and C-reactive protein (CRP). If any of these tests had been conducted within three months before the initial assessment, those values were used as baseline measurements. This approach was used to reflect real-world clinical practice and to minimize participant burden by avoiding unnecessary repeat laboratory testing. Follow-up testing was not required for markers within the normal range, but repeat blood samples were collected at the 3- and 6-month visits if initial results were abnormal.

#### 2.5.4. Maximum Oxygen Uptake Levels (VO_2_ max)

The Submaximal Heart and Pulmonary Evaluation (SHAPE™) is an FDA-approved, noninvasive stress test to estimate VO_2_ max [28]. Participants step on a 5.5-inch platform for 3 min while a simplified gas exchange system monitors heart rate, rhythm, breathing, and gas exchange. The 6-min test includes 2 min of baseline monitoring, 3 min of exercise, and 1 min of recovery. It is considered submaximal because participants are not required to reach VO_2_ max. Participants completed SHAPE™ at baseline and 6 months. Its feasibility, safety, low physical demand, and standardized assessment of functional capacity make it suitable for breast cancer survivors in real-world clinical settings, although it may be less precise than spiroergometry.

### 2.6. Outcomes

The primary objective was to evaluate the feasibility of a six-month hybrid, individualized nutrition and physical activity counseling program for overweight or obese BC survivors to achieve a >10% reduction in baseline body weight. This goal was based on the Women’s Health Initiative Observational Study, which noted that weight loss of >5% may reduce breast cancer risk [29]. This is considered a beneficial and achievable goal. In this pilot study, we assessed whether 10% weight loss over 6 months is also feasible. Weight loss was measured in kilograms at baseline, as well as 3 and 6 months after initiation of intervention.

Secondary endpoints included: (1) changes in body fat percentage; (2) improvement in quality of life; (3) changes in extrapolated VO_2_ max; (4) changes in biomarkers, including the lipid profile (triglycerides, LDL, HDL, and total cholesterol levels), glycemic profile (HbA1c), serum vitamin D (25-hydroxyvitamin D), and serum CRP.

### 2.7. Sample Size and Statistical Analysis

This was a single-arm, non-randomized pilot study. A total of 44 subjects were required to achieve 80% power to detect a 10% reduction in body weight from baseline to 6 months, with a significance level of 0.05. Assuming a dropout rate of 20%, the required sample size was adjusted to 53 participants. To further account for potential attrition, a target enrollment of 60 participants was planned. Participants who did not complete Month 3 assessments could be replaced. Consequently, 78 participants were enrolled over the study period to achieve the target of 60 evaluable participants.

Descriptive statistics, including counts, frequencies, percentages, medians, and interquartile ranges (IQRs; Q1 and Q3), were calculated at baseline and at months 3 and 6. Differences between time points were assessed using Wilcoxon signed-rank tests. *p*-values of <0.05 were considered statistically significant (two-sided). Univariate logistic regression evaluated factors associated with >10% weight loss (odds ratios [ORs] with 95% confidence intervals [CIs]). Analyses were conducted using SAS 9.4 and R 4.2.3.

### 2.8. Use of Generative Artificial Intelligence

**Figure 2:**
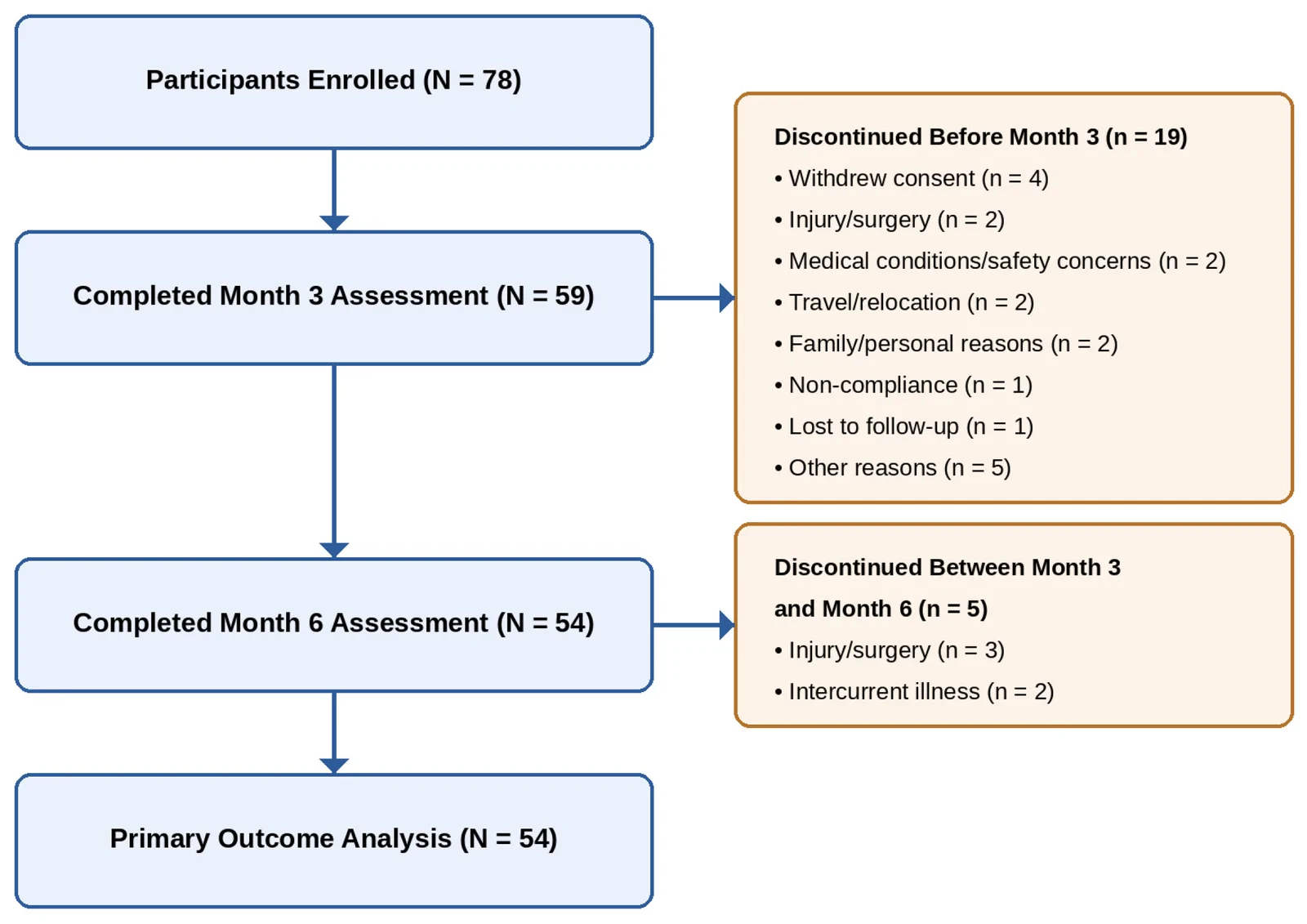
CONSORT-style participant flow diagram. Enrollment and follow-up counts (78 → 59 → 54) and the completer and baseline-observation-carried-forward (BOCF) sensitivity analysis populations (*n* = 54 and *n* = 76, respectively) reflect the study database. Reasons for discontinuation are reported with exact per-reason counts at each time point: before Month 3 (*n* = 19)—withdrew consent (4), injury/surgery (2), medical conditions/safety concerns (2), travel/relocation (2), family/personal reasons (2), non-compliance (1), lost to follow-up (1), other reasons (5); between Month 3 and Month 6 (*n* = 5)—injury/surgery (3), intercurrent illness (2).

During the preparation of this manuscript, the authors used Claude (Anthropic; Claude Opus 5.5 and earlier Claude model versions, accessed via claude.ai) for language editing and copyediting, reorganization of revised text in response to peer-review and editorial comments, consistency checks between the values reported in the text and tables and the statistical output generated by the study team, formatting of tables and references, graphical preparation of the participant flow diagram (Figure 2) from counts supplied by the study team, and typographical corrections in Figure 1. The tool was not used to collect or generate data, perform statistical analyses, interpret the results, or draw conclusions; all statistical analyses were performed by the study team using SAS 9.4 and R 4.2.3. The authors reviewed and edited all output produced with the tool and take full responsibility for the content of this publication.

## 3. Results

Participant flow and retention are summarized in Figure 2.

### 3.1. Participant Flow and Retention

Between October 2021 and November 2023, seventy-eight patients who met the criteria were enrolled in this pilot study. Median program compliance was 4.83 points (IQR 4.17–5.33), with higher scores indicating better adherence to dietary recommendations.

Reasons for discontinuation included withdrawal of consent, surgery or injury, intercurrent medical conditions, relocation or travel, family or personal circumstances, non-compliance, and loss to follow-up.

Compliance scores were also compared across weight-loss outcome categories among the 54 six-month completers. Median compliance scores increased numerically from the weight-gain subgroup (4.67, IQR 3.17–5.00) through the >10% weight-loss subgroup (5.50, IQR 4.83–5.67); this trend did not reach statistical significance (Kruskal–Wallis *p* = 0.0594) and should be interpreted as suggestive rather than confirmatory given the small subgroup sizes.

### 3.2. Baseline Demographic and Clinical Characteristics

The median age was 58.5 years (IQR 13.5); the majority were self-identified as White (72.4%), followed by Black (22.3%) and Asian (4.0%). The median time from cancer diagnosis to enrollment was 882.0 days (IQR 1102.0). For patients who received chemotherapy, the median days from last chemotherapy session date to the enrollment date was 773.0 days (IQR 1073.0). The median weight was 84.4 kg (IQR 19.85); BMI was 31.05 kg/m^2^ (IQR 7.1); and body fat composition was 44.85% (IQR 7.70).

In addition, participants had a median HbA1c of 5.5% (IQR 0.4), a high median LDL level of 108.0 mg/dL (IQR 35.5), and other correlative markers were within normal limits. Participants self-reported to have a moderate quality of life (median FACT-B, 71 points; IQR 19), minimal anxiety (median GAD-7, 2.0 points; IQR 6.5), mild depression (median PHQ-9, 5.0 points; IQR 6.0), low distress (median NCCN Thermometer, 2.0 points; IQR 4.0), and low pain burden, with median BPI pain severity and pain interference scores of 0.75 (IQR 2.38) and 0.29 (IQR 2.36), respectively.

Detailed baseline sociodemographic, anthropometric, and laboratory characteristics are shown in Table 1. The cohort’s key baseline treatment and comorbidity profile is summarized here: 38.5% had received prior chemotherapy, 53.8% were on an aromatase inhibitor and 15.4% on tamoxifen, and the most common comorbidities were hypertension (34.6%) and hyperlipidemia (25.6%); tumor stage was not retained in the study database, although eligibility was restricted to Stage 0–III disease. Complete baseline clinical characteristics are presented in Supplementary Table S1. Longitudinal anthropometric, laboratory, and patient-reported outcome measures at Months 3 and 6 are presented in Table 2.

Variable-specific sample sizes at Month 3 and Month 6 differed substantially. For laboratory measures, sample sizes were as follows: Total cholesterol: *n* = 42 (Month 3), *n* = 36 (Month 6); LDL: *n* = 41 (Month 3), *n* = 36 (Month 6); HDL: *n* = 41 (Month 3), *n* = 36 (Month 6); triglycerides: *n* = 41 (Month 3), *n* = 36 (Month 6); HbA1c: *n* = 26 (Month 3), *n* = 24 (Month 6); serum vitamin D: *n* = 21 (Month 3), *n* = 15 (Month 6); CRP: *n* = 47 (Month 3), *n* = 43 (Month 6); and VO_2_ max: *n* = 52 (Month 6 only).

### 3.3. Primary Outcome: 10% Weight Loss

Thirty-four participants (62.9%) lost ≥1% of their weight. The median weight loss at 3 months was −1.4 kg and −2.71 kg in the final follow-up at 6 months.

At 6 months, 6 participants (11.1%) achieved a weight loss greater than 10%, 10 participants (18.5%) lost between 5% and 10%, 18 (33.3%) experienced a weight loss between 1% and 5%; and 5 (9.2%) had a weight loss of less than 1%; the remaining 15 participants (27.8%) experienced weight gain during follow-up.

Safety outcomes: Medical events leading to study discontinuation included surgery, injury, and other intercurrent medical conditions; however, based on review of available study records, none were attributed to the nutrition and physical activity intervention.

At 6 months, 6 (11.1%) participants achieved the primary outcome of >10% weight loss. Univariate logistic regression for the event of >10% weight loss compared to <10% weight loss showed that the baseline variables were non-significant and were not significantly associated with achieving >10% weight loss.

BMI difference from baseline to 6 months [OR 0.06 (95% CI 0.01–0.53), *p* = 0.0107] and body fat composition difference from baseline to 6 months [OR 0.57 (95% CI 0.37–0.86), *p* = 0.0082] were significantly associated with achieving >10% weight loss. A multivariate regression was not performed due to the small number of participants with the outcome of interest (Table 3).

BMI difference is mathematically related to the >10% weight-loss threshold itself (BMI is a near-linear function of weight at constant height) and should be interpreted as an internal consistency check rather than an independent predictor of response. Body fat composition difference is a correlated but distinct measure and is likewise best interpreted as an expected association rather than a novel finding. Given the small number of outcome events (*n* = 6), both associations should be considered exploratory and hypothesis-generating rather than confirmatory.

### 3.4. Secondary Outcomes: Cardiometabolic Measures (Table 4)

At 6 months, 16 participants (29.6%) achieved >5% weight loss. Univariate logistic regression demonstrated that PHQ-9 difference from baseline to 6 months [OR 0.78 (95% CI 0.63–0.97), *p* = 0.0219], weight difference [OR 0.05 (95% CI 0.004–0.676), *p* = 0.0238], BMI difference [OR 0.01 (95% CI <0.001–0.260), *p* = 0.0058], and body fat composition difference [OR 0.52 (95% CI 0.36–0.77), *p* = 0.0009] were significantly associated with achieving >5% weight loss.

As above, weight difference and BMI difference are mathematically linked to the ≥5% weight-loss threshold itself and should be interpreted as an internal consistency check rather than independent predictors. Body fat composition difference is a correlated but distinct measure and is likewise best interpreted as expected rather than novel. Among the variables tested, only the association with PHQ-9 change, an independent psychological measure with no mathematical relationship to weight, represents a genuinely novel, hypothesis-generating finding. All associations in this analysis should be considered exploratory given the small number of events (16).

Body fat composition decreased significantly in the cohort, with a median change of −0.6% (IQR 2.25; *p* < 0.005) at 3 months and −1.1% (IQR 3.2; *p* < 0.001) at 6 months. Similarly, BMI decreased significantly, with a median change of −0.55 kg/m^2^ (IQR 1.15; *p* < 0.001) at 3 months and −1.10 kg/m^2^ (IQR 1.90; *p* < 0.001) at 6 months.

The median HbA1c decreased by −0.2 (IQR 0.30; *p* = 0.0181) at 3 months, but this change was not sustained at 6 months whereby the change from baseline was −0.05 (IQR 0.55; *p* = 0.5092). The weight in kg decreased by a median of −1.40 (IQR 3.09; *p* < 0.001) at 3 months and by −2.71 (IQR 4.90; *p* < 0.001) at 6 months. Serum vitamin D increased by a median of 10 points (IQR 12.3; *p* = 0.0466) at 3 months with no significant sustained improvement at 6 months [median difference of 5.10 points (IQR 20.10; *p* = 0.5153)]. Surprisingly, the VO_2_ max at 6 months showed a significant median decrease of −1.65 (IQR 7.00; *p* = 0.0315).

**Table 4:** Observed Changes Following the Nutritional Pilot Study Program.

	3 Months		6 Months	
	Median Difference (IQR)	*p*-Value	Median Difference (IQR)	*p*-Value
Primary outcome				
Weight	−1.40 (3.09)	<0.001 *	−2.71 (4.9)	<0.001 *
Secondary outcomes				
BMI	−0.55 (1.15)	<0.001 *	−1.10 (1.9)	<0.001 *
Body Fat Composition	−0.65 (2.25)	0.005 *	−1.10 (3.2)	0.001 *
HbA1c	−0.20 (0.30)	0.0181 *	−0.05 (0.55)	0.5092
Total Cholesterol	−5.00 (27.0)	0.1507	−4.50 (39.0)	0.3291
LDL	−1.00 (21.0)	0.2758	2.50 (30.5)	0.5788
HDL	−2.00 (9.0)	0.1629	−3.00 (9.5)	0.058
Triglycerides	4.00 (25.0)	0.3823	−2.00 (36.0)	0.6415
25-hydroxyvitamin D	10 (12.3)	0.0466	5.10 (20.10)	0.5153
CRP	0.00 (0.2)	0.1682	0.00 (0.4)	0.9443
VO_2_ max	—	—	−1.65 (7.0)	0.0315 *
FACT-B Total score	4.00 (9.17)	0.0001 *	6.00 (11.0)	<0.001 *
Physical well-being	0.00 (3.0)	0.2065	1.00 (5.0)	0.0026 *
Emotional well-being	0.00 (3.0)	0.2201	1.00 (3.0)	0.0458 *
Social well-being	1.00 (4.0)	0.0590	1.00 (4.0)	0.0093 *
Functional well-being	1.00 (4.0)	0.0148 *	1.00 (4.0)	0.001 *
Breast cancer subscale	3.00 (5.0)	<0.001 *	4.00 (6.0)	<0.001 *
GAD-7	0.00 (2.0)	0.0229 *	0.00 (3.0)	0.1771
PHQ-9	−1.00 (3.5)	0.0097 *	−1.00 (4.0)	0.0063 *
BPI-SF				
Pain severity	0.00 (2.0)	0.2090	0.00 (0.75)	0.7822
Pain interference	0.00 (1.07)	0.1801	0.00 (0.71)	0.6435
NCCN Distress Thermometer	0.00 (2.0)	0.6921	0.00 (3.0)	0.9031

* Statistically significant; BMI: body mass index; BPI-SF: Brief Pain Inventory Short Form; LDL: low-density lipoprotein; HDL: high-density lipoprotein; CRP: C-reactive protein; FACT-B: Functional Assessment of Cancer Therapy for Breast Cancer; GAD-7: Generalized Anxiety Disorder 7-item; PHQ-9: Patient Health Questionnaire-9; NCCN: National Comprehensive Cancer Network; HbA1c: hemoglobin A1c; IQR: interquartile range; VO_2_ max: maximum oxygen uptake. —: not assessed at 3 months (VO_2_ max was measured at baseline and 6 months only).

### 3.5. Secondary Outcomes: Quality of Life Questionnaires

At 3 months, depressive symptoms decreased (PHQ-9 median change −1.00, IQR 3.5; *p* = 0.0097), while quality of life improved (FACT-B: 4.00, IQR 9.17; *p* = 0.0001), including functional well-being (1.00, IQR 4.0; *p* = 0.0148) and the breast cancer subscale (3.00, IQR 5.0; *p* < 0.001). A statistically significant difference was observed in GAD-7 scores (median change 0.0, IQR 2.0; *p* = 0.0229). No significant changes occurred in the remaining FACT-B domains, pain severity or interference, or distress.

At 6 months, improvements persisted in depressive symptoms (PHQ-9: −1.00, IQR 4.0; *p* = 0.0063) and quality of life (FACT-B: 6.00, IQR 11.0; *p* < 0.001). Improvements were observed across the physical (1.00, IQR 5.0; *p* = 0.0026), functional (1.00, IQR 4.0; *p* = 0.001), emotional (1.00, IQR 3.0; *p* = 0.0458), and social well-being domains (1.00, IQR 4.0; *p* = 0.0093), as well as the breast cancer subscale (4.00, IQR 6.0; *p* < 0.001). Anxiety, pain, and distress did not significantly change.

Several outcomes demonstrated statistically significant Wilcoxon signed-rank test results despite median differences of 0.00. This occurs because the Wilcoxon signed-rank test evaluates the distribution of paired differences based on their signed ranks rather than the median difference alone. Therefore, a median difference of 0.00 does not necessarily indicate the absence of a statistically significant shift between time points.

## 4. Discussion

In this pilot feasibility study, BC survivors were able to participate in a hybrid (phone and in person) nutritional and physical activity program; participation was associated with significant reductions in weight, body fat percentage, and observed improvements in BMI at both the 3- and 6-month marks. As a prospective, single-arm pilot study, this investigation was not designed to establish definitive efficacy but rather to evaluate feasibility, acceptability, and preliminary clinical outcomes within a real-world survivorship setting. These findings may help inform the design of future randomized studies and support further evaluation of scalable survivorship interventions similar to those examined in the LEAN and LISA trials [30,31]. Additionally, improvements were observed in quality of life. At 6 months, all FACT-B domains showed significant improvement compared to baseline. Improvements in both anxiety and depressive symptoms were observed in the short term, with improvements sustained only for depressive symptoms at 6 months compared to baseline. A modest reduction in HbA1c was observed at 3 months only, with no significant changes in the lipid panel. VO_2_ max showed a significant decline by the end of the study, while pain severity and distress scores showed no significant changes. This study, with its comprehensive outcome framework including VO_2_ max, metabolic and inflammatory biomarkers, and validated psychosocial instruments, facilitates a multidimensional assessment within a real-world survivorship setting.

Obesity is a well-established risk factor for developing BC. It is unclear, however, if patients were overweight/obese before their diagnosis with cancer or if this occurred after treatment. As previously mentioned, weight gain is common during treatment, often related to physical inactivity and dietary changes, especially if chemotherapy is administered [5,6,7]. Although 11.1% of participants achieved the primary objective of >10% weight loss at 6 months, attrition should be considered, as outcomes reflect the 54 participants who completed follow-up out of 78 enrolled at baseline. Although participant attrition occurred during follow-up, retention stabilized after Month 3, with 54 of 59 participants (91.5%) completing the remainder of the study. Notably, 54 participants completed the study, exceeding the sample size required by the original study calculations. Together, these findings support the feasibility of this approach and may help inform future survivorship interventions. The ability to retain more participants than required by the original sample size calculations despite the real-world nature of the intervention further supports the practicality of incorporating similar programs into routine survivorship care. This ≥10% threshold was selected as an exploratory yet clinically meaningful target, building on prior evidence demonstrating that ≥5% weight loss is associated with improved breast cancer outcomes [29].

Furthermore, a significant number experienced >5% weight loss. This was accompanied by a decrease in body fat percentage and an improvement in BMI, which may still confer clinical benefits. Although significant improvements were observed at the cohort level, individual responses were heterogeneous. While 34 participants (62.9%) achieved at least 1% weight loss and 16 (29.6%) achieved >5% weight loss, some participants experienced minimal weight change, and 15 participants gained weight during follow-up. This variability is consistent with prior lifestyle intervention studies in breast cancer survivors [30,31] and highlights the complexity of weight management in survivorship populations. Our findings suggest that while a low-burden counseling program may help a subset of survivors achieve clinically meaningful weight loss, individualized or more intensive strategies may be necessary for others. Participants achieving greater weight loss demonstrated numerically higher compliance scores, although the association did not reach statistical significance (*p* = 0.0594); this pattern is consistent with, though not proof of, a dose-response relationship between dietary adherence and outcome, and warrants confirmation in a larger sample.

Previous research suggests that barriers to healthy eating and physical activity are common among breast cancer survivors [9], and a better understanding of these factors may help identify patients most likely to benefit from low-burden counseling approaches and guide development of more personalized survivorship care strategies.

Previous studies have demonstrated that even modest weight loss could reduce the recurrence of BC [29]. This may be explained by excess adipose tissue being associated with increased circulating estrogen levels [32]. Furthermore, a study evaluating a total of 13,230 patients found that obesity was associated with an increased risk of BC recurrence among postmenopausal patients with hormone receptor-positive, early-stage BC treated with AIs [33]. Therefore, perhaps weight loss might help with the outcomes associated with endocrine therapy. However, this should be interpreted with caution, as the same study found no statistically significant increase in recurrence in patients who were overweight. Therefore, there may be a critical weight when aromatase inhibitors start losing their efficacy.

Previous randomized trials support telephone-based weight-loss interventions in postmenopausal women [30,31].

Although median weight loss in our cohort was approximately 3.2% (−2.71 kg from a baseline median weight of 84.4 kg), compared with 5.4–6.4% in the LEAN trial and 5.3% in the LISA trial, our intervention required substantially less participant contact. Participants in our program completed 7 total contacts (3 in-person and 4 telephone visits), compared with 11 counseling sessions in LEAN and 19 telephone contacts in LISA. This lower-intensity approach may facilitate scalability, reduce patient burden, and facilitate implementation in real-world survivorship care settings. Because the in-person visits were aligned with routine oncology follow-up appointments, additional travel requirements were minimized, potentially reducing transportation burden and improving accessibility for survivors. Given workforce and resource constraints in many survivorship programs, lower-contact interventions may be more feasible to implement widely while still producing clinically meaningful improvements in selected outcomes. Importantly, the intervention was delivered within a real-world survivorship setting rather than under highly controlled trial conditions, potentially increasing the relevance of these findings to routine clinical practice.

The study period overlapped with pandemic-related restrictions on gyms and outdoor activity; although no pandemic-specific variable was collected, this may be relevant context for interpreting activity-related findings. Aligning in-person visits with routine oncology follow-up may have reduced transportation burden and improved access; however, transportation burden, missed wages, financial toxicity, and access were not directly measured in this study.

Among the correlative biomarkers assessed, only HbA1c showed a slight but statistically significant improvement at 3 months, with no sustained changes by the end of the study. This was expected, as most participants had normal baseline HbA1c, limiting the potential for further improvement. Consequently, the opportunity for measurable improvement may have been limited despite modest weight loss and lifestyle changes. In contrast, no notable changes were observed in lipid profiles by study completion. Higher physical activity levels are associated with higher HDL levels [34]. Correlative biomarkers were reassessed at 3 and 6 months only in participants with abnormal baseline values, thus limiting the available data points for comparison and decreasing the statistical power. Additionally, time from cancer diagnosis to enrollment and time from last chemotherapy were unavailable for a substantial proportion of participants (*n* = 39 and *n* = 16 available, respectively), limiting our ability to fully characterize treatment timing within the cohort and potentially affecting interpretation of these descriptive variables.

Because follow-up laboratory testing was obtained only for participants with abnormal baseline values as part of routine clinical care, Table 2 and Table 4 are based on different participant subsets. Table 2 reports cross-sectional laboratory values among participants with available measurements at each time point, whereas Table 4 reports paired within-participant changes among participants with both baseline and follow-up measurements; therefore, the two tables should not be directly compared.

Moreover, evidence suggests that at least 5% weight loss is typically needed to improve lipid parameters [35]. Roughly 29.6% lost at least 5%, but these findings are based on studies in the general population and may not be generalizable to patients with BC. Interestingly, VO_2_ max declined by the end of the study. This result contradicts other studies that reported improvements in VO_2_ max using a standard treadmill test [36]. Our study estimated VO_2_ max using the SHAPE™, which may be less accurate than standard testing. Additionally, it is unclear if some of the discordant results reflect differences in interest and motivation between the beginning of the study, when the test was new and patients were intrigued, and the end, when they were already familiar with it.

Along with weight loss, improvements were observed in quality-of-life measures, including FACT-B. By 6 months, all the FACT-B domains (physical, emotional, social, functional well-being, and the BC subscale) showed improvement compared to baseline. The physical well-being domain reflects patients’ perception of overall health, including fatigue, energy levels, and physical limitations. Previous studies suggest that these symptoms could be improved with exercise training [37].

The published minimal important difference (MID) for the FACT-B is approximately 7–8 points for the total score and 5–6 points for the Trial Outcome Index (TOI) [38]. The observed median FACT-B TOI increase of 6.0 points at 6 months meets this threshold, suggesting the improvement in quality of life may be not only statistically but also clinically meaningful for a substantial portion of participants, although individual-level variability in response should be considered.

Distress remained unchanged, possibly because baseline levels were mild [39,40]. Anxiety improved at 3 months but not at 6 months, whereas depressive symptoms improved throughout follow-up. These findings suggest that clinically meaningful changes may occur despite mild baseline symptoms. Baseline anxiety levels were low, potentially limiting the ability to detect sustained improvement and introducing a floor effect.

Our study has several strengths, including high-quality anthropometric and body composition measurements performed by an RD using bioelectrical impedance. A primarily phone-based design may offer a more scalable and potentially cost-effective approach to oncologic care than more resource-intensive models, though cost-effectiveness, transportation burden, and financial toxicity were not directly measured in this study. While prior studies have used more intensive nutrition and/or exercise interventions, our results indicate that remote counseling was also associated with comparable outcomes, potentially supporting broader implementation. However, some limitations include a small sample size, lack of a control group, and no follow-up beyond six months.

These limitations are consistent with the intended scope of this work as a Stage I feasibility study within a phased approach to behavioral intervention development, such as the ORBIT model, in which single-arm designs are used to establish feasibility and preliminary effect size ahead of an adequately powered randomized controlled trial [41]. This study and its reporting were guided accordingly by the CONSORT extension for randomized pilot and feasibility trials [42], which similarly does not require a concurrent control arm to draw feasibility conclusions. Based on the effect size and variance observed here, a future confirmatory randomized trial would require approximately 130–150 participants per arm to detect a between-group difference of this magnitude with 80% power.

## 5. Limitations

To evaluate the potential for attrition bias, baseline characteristics were compared between the 54 participants who completed 6-month follow-up and the 24 who did not. Most baseline variables, including age, race, ethnicity, weight, BMI, body fat composition, and lipid and glycemic markers, did not differ significantly between completers and non-completers (all *p* > 0.05). However, non-completers had significantly higher baseline PHQ-9 depressive symptom scores (*p* = 0.042), significantly higher BPI pain-interference scores (*p* = 0.042), and significantly lower baseline VO_2_ max (*p* = 0.041) than completers, suggesting that participants with greater baseline psychological and physical symptom burden were somewhat less likely to complete the full program; this should be considered when interpreting the generalizability of the completer-based results. As a sensitivity analysis, outcomes were re-evaluated using baseline-observation-carried-forward (BOCF) imputation for participants who did not complete follow-up (*n* = 76 with baseline weight data available). Under this conservative assumption, 7.9% (6/76) of all participants with baseline weight data achieved the primary outcome of >10% weight loss, compared with 11.1% (6/54) in the completer-only analysis; the absolute number of participants achieving this threshold was unchanged, but the denominator-driven percentage was lower when all enrolled participants were considered. The Wilcoxon signed-rank test for weight change from baseline to 6 months remained statistically significant under BOCF (median difference −0.11 kg, *p* < 0.001), consistent in direction with the completer-based analysis, though attenuated toward zero given the conservative imputation.

Additionally, tumor stage was not retained in the study database and could not be included as a baseline characteristic; given that eligibility was restricted to Stage 0–III breast cancer survivors, the cohort spans a range of disease stages whose distribution cannot be reported, which limits assessment of whether outcomes varied by disease severity.

The potential confounding factors such as fluid retention related to corticosteroid use or lymphedema were not systematically assessed, which may have influenced weight measurements. However, the inclusion of body composition analysis provides complementary insight into changes in adiposity, partially mitigating this limitation. Additionally, VO_2_ max was estimated using a submaximal test, which may be less precise than direct cardiopulmonary exercise testing.

Physical activity data have an important limitation: weekly exercise duration and MET scores described in Section 2.4.2 were intended to be recorded prospectively but were documented in clinical dietitian notes rather than a structured research database, and could not be reliably extracted for quantitative analysis. The physical activity variable available for analysis instead reflects self-reported resistance and aerobic training frequency assessed at baseline, prior to the intervention, and does not capture activity performed during the study period; no significant association was observed between baseline exercise frequency and weight-loss outcome (resistance training *p* = 0.249; aerobic training *p* = 0.898). All physical activity data were self-reported. Exercise recommendations were individualized by the registered dietitian, and access to gyms, exercise classes, and exercise equipment was variably affected by COVID-related restrictions during portions of the study period. Consequently, physical activity recommendations and opportunities for implementation were influenced by resources available to individual participants rather than by a standardized supervised exercise program.

Intervention-period physical activity adherence could therefore not be assessed in this analysis, which we identify as a specific target for structured data capture in future iterations of this program. Table 3 and Table 4 include a substantial number of secondary and exploratory comparisons (approximately 30 across primary, secondary, and exploratory endpoints) that were not adjusted for multiple testing; some statistically significant findings may reflect chance associations, and all secondary and exploratory results should be interpreted as hypothesis-generating rather than confirmatory.

With the emergence of glucagon-like peptide-1 (GLP-1) receptor agonists [43], further research should assess how behavioral weight-management programs such as this one may complement or be sequenced with pharmacologic options within survivorship care, and whether early psychological response (e.g., change in depressive symptoms) could help identify which survivors are most likely to respond to either approach.

Future studies should evaluate survivor-reported barriers, facilitators, adherence, and intervention preferences to better understand differential responses to lifestyle interventions. Such information may help identify patients most likely to benefit from low-burden counseling approaches and guide development of more personalized survivorship care strategies.

## 6. Conclusions

The 6-month hybrid nutrition and physical activity counseling program, delivered by a registered dietitian, was associated with significant reductions in body weight and improvements in quality of life and depressive symptoms among breast cancer survivors. This low-burden intervention, delivered within a real-world survivorship setting, may represent a scalable approach to survivorship care and expand access to counseling across a wider range of clinical settings and patient populations.

## Figures and Tables

**Table 1:** Baseline Sociodemographic, Anthropometric, Laboratory, and Patient-Reported Outcome Characteristics of Participants.

Characteristic	Baseline (*n* = 78)
Age (years), median (IQR) (*n* = 76)	58.50 (13.50)
Race, *n* (%) (*n* = 76)	
White	55 (72.4)
Black	17 (22.3)
Asian	3 (4.0)
Others	1 (1.30)
Ethnicity (*n* = 73)	
Non-Hispanic	50 (68.5)
Hispanic	23 (31.5)
Height (cm), median (IQR) (*n* = 76)	162.60 (10.14)
Weight (kg), median (IQR) (*n* = 76)	84.40 (19.85)
Body Mass Index (kg/m^2^), median (IQR) (*n* = 76)	31.05 (7.10)
Time to enrollment from BC diagnosis, days (IQR) (*n* = 39)	882.0 (1102.0)
Time to enrollment from last chemotherapy, days (IQR) * (*n* = 16)	773.0 (1073.0)
Body Fat Composition (%) (*n* = 76)	44.85 (7.70)
Total Cholesterol (mg/dL), median (IQR) (*n* = 76)	184.50 (40.50)
Triglyceride (mg/dL), median (IQR) (*n* = 76)	95.50 (51.00)
HbA1c (%), median (IQR) (*n* = 75)	5.50 (0.40)
CRP (mg/L), median (IQR) (*n* = 66)	0.45 (0.50)
Physical Well-Being (PWB), median (IQR) (*n* = 75)	24.00 (5.00)
Social Well-Being (SWB), median (IQR) (*n* = 75)	24.00 (7.00)
Emotional Well-Being (EWB), median (IQR) (*n* = 75)	21.00 (5.00)
Functional Well-Being (FWB), median (IQR) (*n* = 75)	22.00 (8.00)
Breast Cancer Subscale (BCS), median (IQR) (*n* = 75)	26.00 (10.00)
FACT-B Trial Outcome Index, median (IQR) (*n* = 75)	71.00 (19.00)
GAD-7, median (IQR) (*n* = 76)	2.00 (6.50)
PHQ-9, median (IQR) (*n* = 76)	5.00 (6.00)
NCCN Distress, median (IQR) (*n* = 76)	2.00 (4.00)
BPI—Pain Severity, median (IQR) (*n* = 76)	0.75 (2.38)
BPI—Pain Interference with daily activities, median (IQR) (*n* = 76)	0.29 (2.36)
LDL (*n* = 76)	108.0 (35.5)
HDL (*n* = 76)	59.0 (20.0)
Vitamin D (*n* = 76)	36.0 (18.8)
VO_2_ max (*n* = 75)	17.9 (6.7)

* Among participants who received chemotherapy. BMI: body mass index; BPI-SF: Brief Pain Inventory Short Form; LDL: low-density lipoprotein; HDL: high-density lipoprotein; CRP: C-reactive protein; FACT-B: Functional Assessment of Cancer Therapy for Breast Cancer; GAD-7: Generalized Anxiety Disorder 7-item; PHQ-9: Patient Health Questionnaire-9; NCCN: National Comprehensive Cancer Network; BC: breast cancer; HbA1c: hemoglobin A1c; IQR: interquartile range; VO_2_ max: maximum oxygen uptake. Note: Variable-specific sample sizes (*n*) are reported directly within the table.

**Table 2:** Longitudinal Characteristics of the Nutrition Pilot Study Participants at Months 3 and 6.

Variable	Month 3 (*n* = 59)	Month 6 (*n* = 54)
Weight (kg), median (IQR)	82.10 (20.10)	79.31 (19.82)
BMI (kg/m^2^), median (IQR)	30.95 (7.05)	30.10 (7.30)
Body Fat Composition (%), median (IQR)	43.45 (8.60)	42.50 (9.60)
Total Cholesterol, median (IQR)	190.50 (59.00)	195.00 (42.50)
LDL, median (IQR)	112.00 (33.00)	118.50 (38.50)
Triglycerides, median (IQR)	98.00 (64.00)	88.00 (47.50)
HDL, median (IQR)	52.00 (22.00)	55.50 (20.50)
HbA1c, median (IQR)	5.90 (0.80)	6.15 (0.80)
Serum Vitamin D, median (IQR)	38.50 (16.30)	35.00 (21.00)
CRP, median (IQR)	0.40 (0.40)	0.50 (0.40)
VO_2_ max, median (IQR)	—	17.15 (5.75)
Physical Well-Being (PWB), median (IQR)	25.00 (5.00)	26.00 (4.00)
Social Well-Being (SWB), median (IQR)	24.00 (6.00)	25.83 (3.67)
Emotional Well-Being (EWB), median (IQR)	21.00 (4.00)	22.00 (5.00)
Functional Well-Being (FWB), median (IQR)	23.00 (8.00)	24.50 (6.00)
Breast Cancer Subscale (BCS), median (IQR)	28.00 (7.00)	30.00 (9.00)
FACT-B Trial Outcome Index, median (IQR)	76.00 (16.83)	80.50 (16.00)
GAD-7, median (IQR)	2.00 (4.50)	1.00 (5.00)
PHQ-9, median (IQR)	3.00 (5.00)	2.00 (5.00)
NCCN Distress, median (IQR)	1.00 (4.50)	1.00 (3.00)
BPI—Pain Severity, median (IQR)	1.38 (3.25)	0.38 (2.25)
BPI—Pain Interference with daily activities, median (IQR)	0.57 (2.93)	0.29 (2.00)

BMI: body mass index; BPI-SF: Brief Pain Inventory Short Form; LDL: low-density lipoprotein; HDL: high-density lipoprotein; CRP: C-reactive protein; FACT-B: Functional Assessment of Cancer Therapy for Breast Cancer; GAD-7: Generalized Anxiety Disorder 7-item; PHQ-9: Patient Health Questionnaire-9; NCCN: National Comprehensive Cancer Network; HbA1c: hemoglobin A1c; IQR: interquartile range; VO_2_ max: maximum oxygen uptake. —: not assessed (VO_2_ max was measured at baseline and Month 6 only).

**Table 3:** Univariate Logistic Regression Analysis for Factors Associated with >10% Weight Loss Compared to <10%.

Variable	OR (95% CI)	*p*-Value
Baseline Variables		
FACT-B TOI	1.07 (0.98–1.16)	0.1181
GAD-7	0.85 (0.63–1.15)	0.2848
PHQ-9	0.86 (0.64–1.14)	0.2821
NCCN	0.88 (0.59–1.31)	0.5171
BPI-PS	0.63 (0.27–1.47)	0.2856
BPI-PI	0.42 (0.08–2.28)	0.3126
Weight	0.94 (0.86–1.02)	0.1542
BMI	0.85 (0.66–1.10)	0.2169
Body Fat Composition	1.05 (0.89–1.23)	0.5602
Total Cholesterol	1.01 (0.99–1.03)	0.4741
LDL	1.01 (0.98–1.04)	0.5144
HDL	1.06 (0.99–1.13)	0.1044
Triglycerides	0.97 (0.93–1.01)	0.1175
HbA1c	0.25 (0.03–1.94)	0.1825
Serum Vitamin D	0.94 (0.86–1.02)	0.1429
CRP	0.78 (0.30–2.05)	0.6165
VO_2_ max	1.06 (0.91–1.23)	0.4493
Difference Variables (6 Months − Baseline)		
FACT-B TOI	1.01 (0.92–1.10)	0.8824
GAD-7	0.99 (0.79–1.23)	0.8922
PHQ-9	0.95 (0.77–1.17)	0.6021
NCCN	0.95 (0.70–1.29)	0.7543
BPI-PS	0.94 (0.62–1.42)	0.7520
BPI-PI	1.02 (0.62–1.66)	0.9497
Weight	0.04 (<0.001–2.28)	0.1157
BMI	0.06 (0.01–0.53)	0.0107 *
Body Fat Composition	0.57 (0.37–0.86)	0.0082 *
Total Cholesterol	1.02 (0.98–1.05)	0.3976
LDL	1.02 (0.98–1.06)	0.4152
HDL	1.01 (0.88–1.15)	0.9101
Triglycerides	1.01 (0.98–1.04)	0.6262
HbA1c	21.60 (0.30–>999.99)	0.1596
Serum Vitamin D	1.01 (0.96–1.06)	0.8162
CRP	1.31 (0.49–3.53)	0.5907
VO_2_ max	1.01 (0.86–1.20)	0.8891

* Statistically significant; OR: odds ratio; CI: confidence interval; TOI: Trial Outcome Index; BPI-PS: BPI pain severity; BPI-PI: BPI pain interference; BMI: body mass index; BPI-SF: Brief Pain Inventory Short Form; LDL: low-density lipoprotein; HDL: high-density lipoprotein; CRP: C-reactive protein; FACT-B: Functional Assessment of Cancer Therapy for Breast Cancer; GAD-7: Generalized Anxiety Disorder 7-item; PHQ-9: Patient Health Questionnaire-9; NCCN: National Comprehensive Cancer Network; HbA1c: hemoglobin A1c; VO_2_ max: maximum oxygen uptake.

## Data Availability

The datasets generated and/or analyzed during the current study are available from the corresponding author upon reasonable request.

## Supplementary Materials

The following supporting information can be downloaded at: https://www.mdpi.com/article/10.3390/cancers18193198/s1, Table S1: Baseline Clinical Characteristics of Participants (*n* = 78).
