# Supplementary material for: Quality-of-Life and Weight Loss Outcomes Following an Individualized Nutrition and Physical Activity Counseling Program in Breast Cancer Survivors: A Prospective Single-Arm Pilot Study
Source: Cancers (Basel). 2026 Oct 3;18(19):3198. doi: 10.3390/cancers18193198 (PMC13664228; doi:10.3390/cancers18193198)
Supplement: Supplementary file 1 [file cancers-18-03198-s001.zip › cancers-4547943-supplementary.pdf]

Supplementary Materials

Table S1. Baseline Clinical Characteristics of Participants (N = 78).

Characteristic	n (%)
Prior chemotherapy*	30 (38.5%)
**Endocrine therapy**	
Aromatase inhibitor (anastrozole/letrozole/exemestane)	42 (53.8%)
Tamoxifen	12 (15.4%)
Other endocrine therapy (goserelin only)	2 (2.6%)
No documented endocrine therapy	22 (28.2%)
**Comorbidities**	
Hypertension	27 (34.6%)
Hyperlipidemia / Hypercholesterolemia / Dyslipidemia	20 (25.6%)
Diabetes mellitus / Type 2 DM	7 (9.0%)
Prediabetes	1 (1.3%)
Hypothyroidism / Thyroid dysfunction	9 (11.5%)
Osteopenia / Osteoporosis	10 (12.8%)
GERD / Reflux / Acid reflux	7 (9.0%)
GLP-1 receptor agonist use (Semaglutide/Ozempic)	1 (1.3%)

Tumor stage was not retained in the study database; however, eligibility was restricted to Stage 0–III breast cancer survivors.
Note: Medication and comorbidity data were abstracted from the available study records and reflect the variables included in the study database.
